# Size-Exclusion Chromatography Separation Reveals That Vesicular and Non-Vesicular Small RNA Profiles Differ in Cell Free Urine

**DOI:** 10.3390/ijms22094881

**Published:** 2021-05-05

**Authors:** Jenni Karttunen, Sarah E. Stewart, Lajos Kalmar, Andrew J. Grant, Fiona E. Karet Frankl, Tim L. Williams

**Affiliations:** 1 Department of Veterinary Medicine, University of Cambridge, Cambridge CB3 0ES, UK; jmk78@cam.ac.uk (J.K.); lk397@cam.ac.uk (L.K.); ajg60@cam.ac.uk (A.J.G.); 2 Metabolic Research Laboratories, Wellcome Trust-Medical Research Council Institute of Metabolic Science, University of Cambridge, Cambridge CB2 0QQ, UK; S.Stewart@latrobe.edu.au; 3 Department of Biochemistry and Genetics, La Trobe Institute for Molecular Science, La Trobe University, Bundoora, VIC 3086, Australia; 4 Department of Medical Genetics, University of Cambridge, Cambridge CB2 0QQ, UK; fek1000@cam.ac.uk

**Keywords:** urine, extracellular vesicles, small RNA

## Abstract

Urinary extracellular vesicles (EVs) and their RNA cargo are a novel source of biomarkers for various diseases. We aimed to identify the optimal method for isolating small (<200 nm) EVs from human urine prior to small RNA analysis. EVs from filtered healthy volunteer urine were concentrated using three methods: ultracentrifugation (UC); a precipitation-based kit (PR); and ultrafiltration (UF). EVs were further purified by size-exclusion chromatography (SEC). EV preparations were analysed with transmission electron microscopy (TEM), Western blotting, nanoparticle tracking analysis (NTA) and an Agilent Bioanalyzer Small RNA kit. UF yielded the highest number of particles both before and after SEC. Small RNA analysis from UF-concentrated urine identified two major peaks at 10–40 nucleotides (nt) and 40–80 nt. In contrast, EV preparations obtained after UC, PR or SEC combined with any concentrating method, contained predominantly 40–80 nt sized small RNA. Protein fractions from UF+SEC contained small RNA of 10–40 nt in size (consistent with miRNAs). These data indicate that most of the microRNA-sized RNAs in filtered urine are not associated with small-sized EVs, and highlights the importance of removing non-vesicular proteins and RNA from urine EV preparations prior to small RNA analysis.

## 1. Introduction

Extracellular vesicles (EVs) are membrane-bound particles which have many functions, including intercellular communication, immunomodulation and regulation of angiogenesis. They carry various cargo, including non-coding RNA (ncRNA), messenger RNA (mRNA), proteins, lipids and metabolites [1]. EVs reflect the status of the secreting cell and their use as a ‘liquid biopsy’, whereby EV cargo is analysed instead of tissue samples, is gaining interest as a diagnostic tool [2,3]. MicroRNAs (miRNAs) are an extensively studied small ncRNA subgroup, which regulate gene expression at the post-transcriptional level by binding to target mRNAs [4]. Urinary EV (uEV) based miRNA biomarkers have been investigated in several diseases, including prostate and bladder cancer, and Immunoglobulin A nephropathy [5,6,7,8,9]. Despite their potential, translation of EV-based biomarkers into clinically tractable diagnostic tests has been challenging [10].

To date, studies investigating uEV-based small RNA biomarkers have focused on miRNAs. However, miRNAs constitute a relatively small percentage of total small RNA present in uEVs and urine, and the uEV preparations contain large amounts of other small RNAs, including tRNA and YRNA fragments [11,12,13]. For example, in uEV samples, miRNAs constituted 19–33% of the raw reads depending on the library preparation method and YRNA 54–66% of the other ncRNA reads [12]. In urine sequencing studies, miRNAs have constituted 12.8% and tRNA 84% of mapped reads [11] and 2% and 11%, respectively, of the raw reads [13]. Extracellular small RNAs are carried by EVs and other non-vesicular components, including lipoproteins and ribonucleoprotein particles (RNPs), such as argonaute 2 (AGO2) [14,15,16,17]. Most of the aforementioned studies have investigated the distribution of small RNAs in plasma; however, it could be postulated that some small RNAs found in impure uEV preparations are also not within, or associated with, EVs. The efficacy of different EV purification methods to separate EVs from other RNA carriers is variable [18,19], which might explain why the RNA profiles of EVs vary between studies that utilise different EV purification methods [20,21,22]. The use of EV purification methods which yield the purest EV preparations will increase the likelihood of identifying suitable EV-based small RNA biomarkers; however, methods that optimise EV purity often reduce EV yield, which in turn could decrease test sensitivity.

Different EV isolation methods have been compared and evaluated in several review articles [23,24]. Ultracentrifugation (UC) is often considered the ‘gold standard’ for EV purification; however, this method can yield impure preparations and requires expensive equipment [23]. Several commercial polymer-based precipitation kits (PR) have been introduced which facilitate the isolation of EVs using standard laboratory centrifuges; however, they also yield impure preparations [24,25]. Size-exclusion chromatography (SEC) is gaining more interest as a method to separate EVs from non-vesicular proteins, although it does not separate similar-sized lipoproteins from EVs, which complicates its use when working with plasma [25,26]. Co-isolation of lipoproteins with EVs should be less problematic when working with urine since this biofluid contains few lipoproteins [27]. Therefore, analysis of the RNA profile of different SEC fractions (EV fraction and non-vesicular protein fraction) should allow comparison of the RNA profiles of urine EVs and non-vesicular carriers.

The primary aim of this study was to analyse the small RNA profile of small uEVs obtained by different methods. Aliquots of 0.22 μm filtered urine were concentrated by ultracentrifugation (UC), precipitation (PR) and ultrafiltration (UF) prior to further purification by SEC. The small RNA profile of UF-concentrated urine (containing both EVs and proteins >100 kDa) was compared to the profile of the EV and protein fractions obtained after SEC. A secondary aim of the study was to determine the optimal method to concentrate uEVs before SEC purification, and identify if further SEC purification increases the purity of uEVs prepared by these methods.

## 2. Results

### 2.1. Comparison of Particle and Protein Yield between Methods

**Figure 1:**
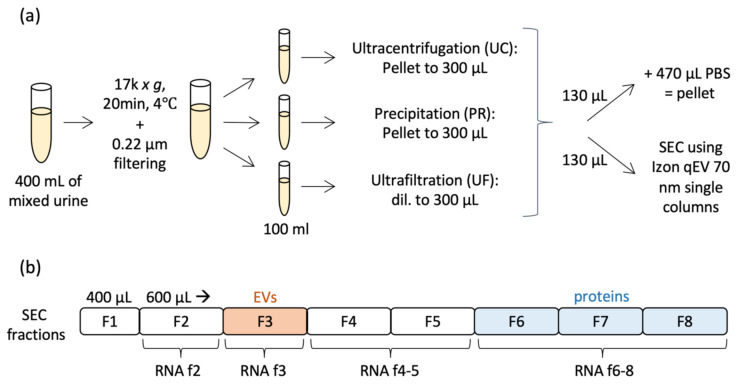
Schematic representation of the study. The protocol was repeated three times. (**a**) After concentrating and filtering, the mixed urine pool of two donors was divided into three aliquots. Each aliquot was concentrated by ultracentrifugation (UC), precipitation (PR) or ultrafiltration (UF). Half of the concentrated urine was analysed as a “pellet” sample and the other half was further purified with size exclusion chromatography (SEC). (**b**) During SEC, eight fractions were collected. Small RNA analysis was performed on SEC fractions 2 (“pre EVs”), 3 (“EVs”), combined fractions 4–5 (“middle”) and 6–8 (“non-vesicular proteins”).

We compared the particle and protein yield obtained using three EV concentrating methods (UC, PR and UF), both before and after SEC. The experimental procedure presented in Figure 1 was repeated three times.

**Figure 2:**
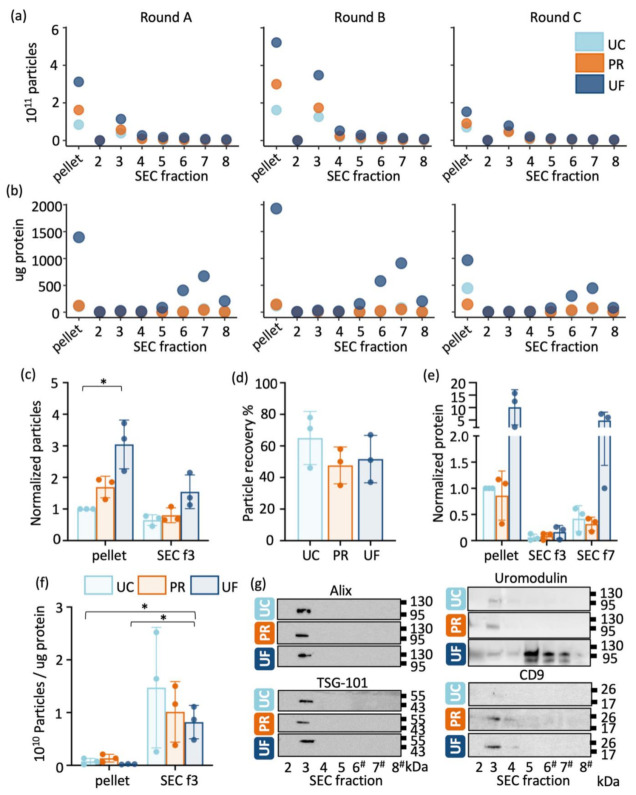
Comparison of EVs concentrated using three different methods (“pellet”) and following SEC purification: (**a**) Particle number measured with nanoparticle tracking analysis (NTA) and (**b**) protein amount from pellet and SEC fractions 2–8 from individual rounds. (**c**) Particle numbers from pellet and SEC fraction 3 (EVs) samples normalised to that of UC pellet in that round. (**d**) Particle recovery calculated by comparing particle yield between fraction 3 and corresponding pellet. (**e**) Protein concentration of pellets and SEC fractions 3 (EVs) and 7 (fraction with highest protein content) normalised to that of the UC pellet in that round. (**f**) Particle/protein ratio calculated from each pellet and SEC fraction 3. With UC+SEC and PR+SEC, the protein concentration was below the sensitivity limit of the assay and the values were estimated as described in the Materials and Methods section. Statistical comparison was not performed against estimated values. (**g**) Western blot analysis of vesicle markers Alix, TSG-101 and CD9 and the contaminating protein uromodulin in SEC fractions 2–8. # Because of high protein concentration, ultrafiltration fractions 6–8 were loaded with 5 μL of the elute instead of 20 μL for other samples. Data are presented as individual values, mean and standard deviation. * *p* < 0.05.

Particle numbers (measured with NTA) and protein quantity within pellets and SEC fractions 2–8 from individual rounds are presented in Figure 2a,b. Particle number peaked within SEC fraction 3, indicating the presence of EVs, and the highest protein quantities were detected in fractions 5–8. Within each replicate, UF resulted in the highest number of vesicles in both the pellet and SEC fraction 3 groups. Since the three replicates were derived from different donors, and thus would contain different quantities of EVs and proteins, the particle number and protein quantity for each replicate was normalised to that of the UC pellet for that replicate, prior to statistical comparison. The normalised mean particle number of the UF pellet was approximately three times that of the UC pellet (*p* = 0.01) and 1.8 times that of PR pellet, although the latter did not reach statistical significance (*p* > 0.05) (Figure 2c). The normalised mean particle number of the PR pellet was approximately 1.7 times that of the UC pellet, although this did not reach statistical significance (*p* > 0.05). After SEC, UF+SEC had the highest normalised particle number (mean 1.55), with PR+SEC and UC+SEC yielding mean normalised particle numbers of 0.80 and 0.65, respectively; however, these differences were not statistically significant. Particle recovery within fraction 3 after SEC (compared with that of the corresponding pellet) was between 46% and 78% for UC, between 35% and 58% for PR, and between 37% and 67% for UF (Figure 2d). 

When the normalised mean protein content of the pellets was compared between methods, the UF pellet contained approximately 10 times more protein than the UC pellet, and the PR pellet contained approximately 15% less protein than the UC pellet (Figure 2e). It should be noted that the UC protocol used in the present study did not include a washing step; therefore, the amount of non-vesicular protein is likely to be higher than the amount yielded if an additional wash step was incorporated into the method. After SEC, the highest protein concentrations were found in fraction 7 (corresponding to non-vesicular proteins) in all samples; however, a small peak was also observed in SEC fraction 3, likely corresponding to EV-associated proteins. SDS-PAGE analysis from individual rounds showed variable protein profiles within pellet samples obtained using the three concentrating methods and almost identical protein profiles after SEC with all three concentrating methods (Supplementary Figure S1). This further indicates the presence of contaminating proteins in the pellet samples.

The purity of vesicles in each sample was further evaluated using the particle/protein ratio (Figure 2f). For UC+SEC and PR+SEC, the particle/protein ratio could not be calculated precisely, because the protein concentration of these samples was below the limit of reliable detection. The particle/protein ratios calculated based on the assigned arbitrary values are shown in Figure 2f. The UF pellet had the lowest particle/protein ratio with mean of 2.2 × 10^8^ particles/μg protein (Figure 2f). The UC and PR pellets had a mean particle/protein ratio of 7.4 × 10^8^ and 1.4 × 10^9^ particles/μg protein, respectively. UF+SEC samples had a significantly greater particle/protein ratio (8.2 × 10^9^ particles/μg protein) than the UC and UF pellets (*p* = 0.03 and *p* = 0.01, respectively).

Western blot analysis verified the presence of the common EV proteins Alix, Tsg-101 and CD9 in SEC fraction 3 from all samples, thus confirming EV isolation (Figure 2g, Supplementary Figure S2). Uromodulin, which is considered the main contaminating protein in urine [18], was mostly present within fractions 6–8 in the UF+SEC, although a small amount was also present within fraction 3 in all samples, which may reflect EV-associated uromodulin.

### 2.2. Transmission Electron Microscopy Reveals Heterogeneous Vesicle Populations

**Figure 3:**
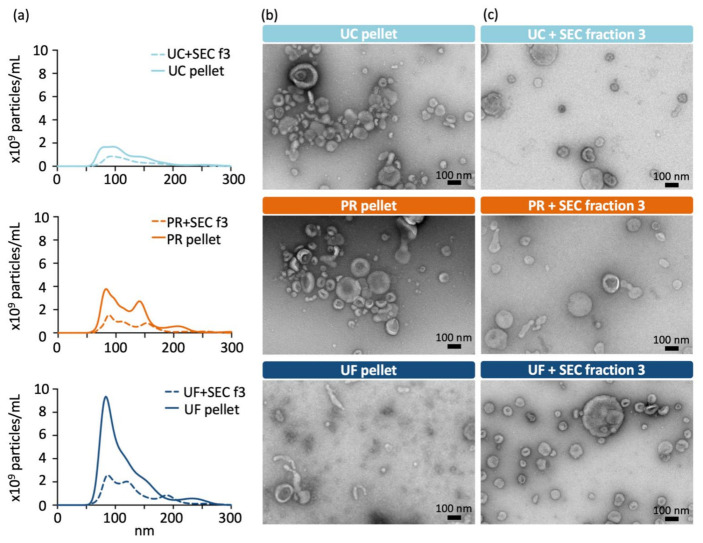
Characterisation of EV size profiles and morphology: (**a**) Particle size distributions measured with nanoparticle tracking analysis (NTA) from ultracentrifugation (UC), precipitation-based kit (PR) and ultrafiltration (UF) samples before and after size-exclusion chromatography (SEC) purification. Mean of three 60 s recordings is shown. Transmission electron microscopy (TEM) images of purified or concentrated EVs (**b**) before and (**c**) after the SEC purification.

The size distribution of EVs obtained from a single urine pool by the different methods are shown in Figure 3a. Size profiles before and after SEC differed the most in the UF sample, probably due to the removal of (smaller) contaminant particles during the SEC. Similar but smaller shifts in the size profiles were also visible after SEC with UC and PR samples. Transmission electron microscopy (TEM) imaging confirmed that the UF pellet contained large amounts of non-vesicular material, as expected (Figure 3b). UC and PR pellets and all the SEC fraction 3 samples included a heterogeneous population of vesicles, many of which demonstrated a cup-shaped morphology that is expected for EVs in TEM (Figure 3b,c) [28,29].

### 2.3. Enriched Extracellular Vesicles Contain Mainly 40–80 nt Small RNA

Differences in the small RNA profiles were evaluated using the Agilent Bioanalyzer small RNA chip. We confirmed that the small RNA profile was similar in samples that were and were not subjected to an initial low speed centrifugation (10 min at 1000× *g*) prior to centrifugation at 17,000× *g* (Supplementary Figure S3); therefore, significant contamination of urine samples by RNA released from ruptured cells during centrifugation at 17,000× *g* was not apparent when fresh urine samples were used.

**Figure 4:**
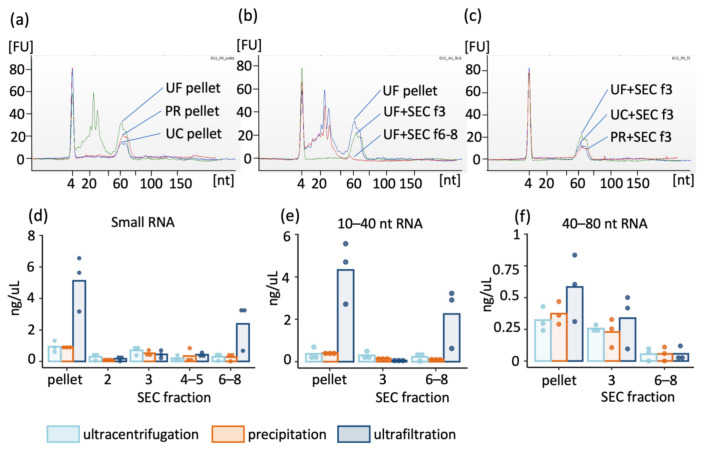
Small RNA profiles of extracellular vesicles (EV) prepared by ultrafiltration (UF), precipitation (PR) and ultracentrifugation (UC) both before and after size-exclusion chromatography (SEC). Representative profiles of samples from individual round of: (**a**) the pellets before SEC purification, (**b**) UF profile before SEC, EV containing SEC-fraction 3 and non-vesicular protein fractions 6–8, (**c**) EV containing SEC fraction 3 from all three methods. RNA concentration from all three rounds showing: (**d**) Total small RNA concentration in measured fractions, (**e**) 10–40 nt sized RNA in UF pellets and SEC fractions 3 (EVs) and 6–8 (non-vesicular proteins), (**f**) 40–80 nt sized RNA in UF pellets and SEC fractions 3 and 6–8. Data are presented as individual values and mean.

Small RNA profiles were obtained for each pellet and SEC fractions 2, 3, 4–5 combined and 6–8 combined. The RNA profile of UC and PR pellets included one major peak at 40–80 nt, whereas the UF pellet (representing that of concentrated urine) contained two major peaks: the first at 10–40 nt and the second at 40–80 nt (Figure 4a). After the UF pellet was further purified by SEC, the EV-containing fraction 3 yielded a single major peak at 40–80 nt and the non-vesicular protein-containing fractions 6–8 yielded a peak at 10–40 nt (Figure 4b). Regardless of the initial concentrating method used, the small RNA profile of the EV-containing fraction 3 was similar after SEC, with a main peak at 40–80 nt (Figure 4c). The majority of the total small RNA was present within SEC fractions 3 and 6–8 after UF, and in fraction 3 after PR and UC; however, significant differences between groups were not apparent (Figure 4d). As expected, fractions 2, 4 and 5 contained low quantities of small RNA (Figure 4d). The majority (64–86% of the total amount in all SEC fractions) of 10–40 nt-sized small RNA in samples concentrated by UF was present within fractions 6–8, with smaller quantities (0–9% of the total amount in all fractions) detected in fraction 3 (*p* > 0.05; Figure 4e). Reliable calculation of the percentage of 10–40 nt sized small RNA within fractions 3 and 6–8 from samples concentrated by UC and PR was not possible due to the low quantities of 10–40 nt sized RNA present. The low quantity of 10–40 nt sized small RNA within these samples likely reflects removal of the majority of proteins (and their associated 10–40 nt sized RNA) during the UC and PR purification. The 40–80 nt-sized small RNA was observed in all pellets, and following SEC, a large proportion (42–75%) of the 40–80 nt-sized small RNA was evident within fraction 3, with significantly smaller quantities (0–26%) observed in fractions 6–8 (*p* = 0.004), regardless of the initial concentrating method used (Figure 4f).

## 3. Discussion

Our results indicate that the majority of microRNA-sized (10–40 nt) small RNAs in 0.22 μm filtered urine are neither within nor associated with small EVs, whereas the longer 40–80 nt sized small RNAs co-purify with small EVs. The UF concentrated urine RNA profile (representing concentrated 0.22 μm filtered urine) included two main peaks: one sized 10–40 nt and another sized 40–80 nt, which is consistent with the previously reported small RNA profiles obtained from urine [30]. After SEC, these two peaks were identified within different fractions; most 10–40 nt RNA (including miRNA) in UF concentrated samples was identified within non-vesicular protein fractions, whereas the 40–80 nt sized RNA was found in the (small) EV-containing SEC fraction 3. Furthermore, the small RNA profiles of EV preparations obtained by UC and PR also predominantly contained 40–80 nt sized RNA, with minimal 10–40 nt sized RNA being detected. Previously, Lozano-Ramos and others used SEC and, similar to our study, reported a 60 nt small RNA peak from the EV fraction [31]. Gheinani et al. used NanoString nCounter to measure miRNAs from total urine and UC+SEC purified uEVs [32]. Out of the 800 miRNAs probed, they were able to stably detect 256 miRNAs from at least one sample. However, only 18 miRNAs were present in all 6 uEV samples, whereas 41 miRNAs were detected from all corresponding total urine samples. These data support our findings that suggest that relatively small amounts of miRNAs in filtered urine are present within, or associated with, small EVs. However, our study did not attempt to evaluate the 10–40 nt sized RNA content of large-sized EVs, which could potentially contain more miRNA sized RNAs than small EVs. Therefore, further studies to evaluate the RNA content of large EVs isolated by SEC are warranted. Although most miRNA-sized small RNA in filtered urine was found to be associated with non-vesicular fractions, data from other studies indicate that some miRNA can be identified in the EV-containing SEC fractions when using more sensitive methods such as quantitative PCR or next generation sequencing [32,33]; thus, EVs should still be considered to contain, or be associated with, miRNAs. It is also possible that the miRNA packaging to EVs is altered and especially increased in certain disease conditions. However, our data emphasize the importance of EV purification and robust separation of EVs from non-vesicular proteins, particularly if specific analysis of EV-associated small RNA content is desirable. When attempting to identify small RNA biomarkers, evaluation of the small RNA content of specific SEC fractions, for example the EV-containing SEC fraction 3, rather than the total extracellular small RNA content, might decrease the background variability and increase the possibility of finding a robust biomarker. 

The exact carrier of the small RNA in the non-vesicular protein fractions was not investigated in this study. Earlier studies have shown that the vast majority of plasma miRNAs are in the non-vesicular SEC fractions, associated with AGO2 instead of EVs [16,17]. AGO2 is also present in urine, and at least miR-16 and miR-192 have been found to be associated with the AGO2 complex [34]. New evidence is also emerging about even smaller (<50 nm) non-membranous nanoparticles which are enriched with Argonaute 1–3 proteins and could, in theory, elute in the late SEC fractions [35]. However, the EVs concentrated by UC and PR only (i.e., the “pellet” samples) had similar small RNA profiles to the EV fractions obtained after SEC, thus suggesting that these techniques also excluded the carrier of the majority of the 10–40 nt sized small RNA. Given that these techniques (especially PR) will not specifically exclude small (<50 nm) EVs, it could be speculated that the carriers of 10–40 nt sized small RNAs are non-vesicular. This hypothesis is supported by the Western blot data, which did not show the presence of canonical EV markers in the late SEC fractions.

Our analysis did not reveal what subtype of small RNA is included in the 40–80 nt peak that is associated mainly with small EVs obtained after SEC. The profile observed in our study is similar to that of mature tRNA molecules, which are ~75–80 nt in size [36] although mature YRNAs are close to the same size range (80–120 nt) [37]. Next generation sequencing could be utilised to further investigate the small RNA cargo of SEC purified uEVs; however, it has technical challenges. For example, biases related to adapter ligation, adapter dimerisation and uneven PCR amplification during small RNA sequencing library preparation have been identified between protocols [38,39]. Furthermore, generally used transcriptase enzymes are not capable of processing highly complex RNA structures, including mature tRNA [40]. Furthermore, many of the small RNAseq library preparation methods are optimized for miRNA analysis and size-selecting for molecules shorter than 40 nt [12,39]. It seems that the 40–80 nt sized molecules, which dominate the small uEV cargo, are not included in the majority of small RNAseq studies published to date. Interestingly, some studies have used alternative sequencing methods and have found a greater amount of mature tRNA molecules from cell culture EVs and plasma [41,42,43], similar studies have not been performed on uEVs to date.

When the three concentrating methods prior to SEC were compared, UF yielded the highest number of vesicles (based on NTA data). These results are not surprising, given that UF simply concentrates the urine, and NTA can also quantify non-EV particles, including larger protein aggregates [44,45]. However, the presence of a higher quantity of non-EV particles may not fully explain the differences between the concentrating methods, as a numerically higher number of particles was found with UF also after further purification with SEC. In support of this, UF following density gradient centrifugation has been shown to yield more vesicles compared to UC following density gradient centrifugation [28]. Furthermore, if it is assumed that EV recovery after SEC is roughly constant regardless of the initial concentration method used, then comparison of particle concentrations in the SEC fraction 3 between methods suggests that concentrating the sample by PR or UC results in approximately a 50% loss of EV-sized particles. These data are supported by an earlier study which suggests that a subgroup of up to 40 % of total uEVs remain in the supernatant after UC at 200,000 g [46]. Previous comparison of five EV isolation methods from urine showed that UC+SEC yielded similar number of vesicles compared to UF+SEC. However, the study used PES-based filters (which might trap more EVs) in ultrafiltration [32], whereas our study used regenerated cellulose membrane filters. The particle recovery after SEC (based on NTA data) was 35–78% with all concentrating methods, indicating some loss of vesicles during the purification. However, the reduction in particle yield, as determined by NTA, observed after SEC could reflect both a loss of non-vesicular particles and a loss of vesicles.

The particle/protein ratio of SEC fraction 3 was higher than the corresponding pellet in UF samples, as expected. Accurate comparison of the particle/protein ratios of SEC fraction 3 obtained after PR and UC was not possible, because the protein concentration of the PR+SEC and UC+SEC samples was below the accurate limit of detection. However, our data suggest that the purity of these samples was at least similar to that of UF+SEC samples. In the present study, the particle/protein ratio of PR pellets (prior to SEC) was significantly greater than that of UC pellets; however, the UC protocol we used did not incorporate a wash step because we aimed to maximise particle yield, rather than purity, prior to SEC. Therefore, it is likely that the purity of samples obtained by UC would be higher (and particle yield would likely be lower) if an additional wash step was used. However, earlier studies have also reported a relatively low purity of the EVs prepared by UC even when a wash step was incorporated [28,47].

One limitation of the present study was that RNAse inhibitors were not used. RNA is extremely prone to degradation especially in the extracellular space, and although EVs are considered to protect their RNA cargo, the small RNAs attached to the outside of EVs may be less protected. Previously, it was reported that adding RNAse inhibitor to the sample at an early stage helps to improve the integrity of the non-vesicular RNA, and interestingly, the authors noted that some of the small extracellular RNAs detected can be stable products of extracellular degradation instead of secretory products of the cells [48]. To maximise the quantity and integrity of RNA in the samples, addition of a RNAse inhibitor before processing could be beneficial and this could be investigated in future studies. Further studies could also be considered to investigate confounding factors that might influence the efficiency of EV isolation from urine. For example, Tamm-Horsfall protein can form a filament network that binds miRNAs and can interfere with EV isolation, and this effect can be exacerbated by acidic pH or presence of Ca^2+^ or Na^+^ ions; hence, several methods have been developed to address the problem [49,50,51]. Here, we used the same urine pool for all methods in each individual round to exclude the effect of these confounding factors. However, additional studies are warranted to confirm if the performance of the different methods are influenced by factors such as urine pH.

In summary, based on our comparison of 50 mL urine aliquots, UF followed by SEC yields a similar number of particles compared to PR, and a higher number of particles than UC and SEC preparations prepared after concentration of the sample by either PR or UF. The purity of the EV preparations prepared by SEC following UF tended to be greater than those prepared by PR alone, and was significantly greater than those prepared by UC alone. The major disadvantage of UF is that it can be time- and labour-intensive, especially with larger starting volumes and a high number of samples. Therefore, when maximal EV recovery is not required, PR might be a more convenient way to concentrate the particles prior to the SEC. Small RNA analysis indicated that the majority of small RNA in urine are not associated with small EVs, and it is likely that mature tRNA-sized RNAs are the most abundant RNA associated within small uEVs of healthy humans. Most miRNA-sized small RNAs were associated with non-vesicular proteins, thus highlighting the importance of EV purification and robust separation of EVs from non-vesicular proteins if specific analysis of small EV-associated small RNA content is desirable; however, further studies of the RNA content of large sized EVs are warranted.

## 4. Materials and Methods

### 4.1. Urine Collection and Pre-Processing

Urine samples were collected from healthy volunteers under Cambridgeshire Research Ethics Committee approval (08-H0306-62) and with the informed consent of participants. Fresh, first void, urine samples without urine dipstick abnormalities (Siemens Multistix 10SG, no proteinuria, haematuria, glucosuria or leukocyte positive reactions detected) were used. Subsequently, the urine from two different donors were combined to obtain 400 mLs of urine (i.e., three replicates, from six healthy donors in total). Eight protease inhibitor tablets (#A32963, Thermo Scientific™, Waltham, MA, USA) were dissolved into each combined sample before the urine was processed. Since the focus of the study was on the study of small uEVs, fresh urine was centrifuged (17,000× *g*, 20 min, +4 °C) and filtered (0.22μm filter, #S2GPU05RE, Millipore, Burlington, MA, USA) in order to remove cells and bacteria; therefore, larger vesicles (>220 nm) would have been mostly excluded from the downstream analyses. The filtered urine was divided into 100 mL aliquots and used to concentrate EVs via one of three different methods: ultracentrifugation (UC), precipitation (PR) and ultrafiltration (UF).

### 4.2. Ultracentrifugation

Two 50 mL aliquots of filtered urine were diluted to 65 mL with filtered phosphate buffered saline (PBS) and EVs were pelleted by UC (Beckman Optima XE ultracentrifuge, Ti45 rotor, 235,400 g, 2 h, +4 °C, acceleration setting: max, deceleration setting: 7). After removing the supernatant by pipetting, individual EV pellets were re-suspended in 100 μL of 0.22 μm filtered PBS, combined, and the total volume was adjusted to 300 μL. A 130 μL aliquot of the concentrated EVs was taken, diluted to 600 μL with 0.22 μm filtered PBS and named “UC-pellet”. Another 130 μL aliquot was used for SEC. As our main aim was to concentrate the maximum number of vesicles prior to SEC, we did not wash the pellet and perform a second ultracentrifugation step.

### 4.3. Precipitation

Four 25 mL aliquots of filtered urine were combined with 10 mL of precipitation buffer (miRCURY Exosome Cell/Urine/CSF Kit, #76743, Qiagen, Hilden, Germany) and placed on a roller mixer overnight (o/n) (16–18 h) at +4 °C. The following day, EVs were pelleted using centrifugation (3200× *g*, 30 min, at room temperature (RT)), the supernatant was removed and an additional 5-min centrifugation was performed to allow removal of the residual supernatant. The pellets in individual tubes were re-suspended in 50 μL of resuspension buffer provided with the kit, combined together, and the total volume was adjusted to 300 μL with 0.22 μm filtered PBS. A 130 μL aliquot of the concentrated EVs was taken, diluted to 600 μL with 0.22 μm filtered PBS and named “PR-pellet”. Another 130 μL aliquot was used for SEC.

### 4.4. Ultrafiltration

Amicon ultra concentrators (100 kDa cut off, #UFC810024, Millipore, Burlington, MA, USA) were washed with 5 mL of filtered PBS (5 min, 4000× *g*, RT), before 15 mL of filtered urine was placed in two columns and centrifuged (10 min, 4000× *g*, RT). The flow through was discarded and the procedure was repeated until the sample was concentrated to <250 μL. The concentrated urine was adjusted to 300 μL with 0.22 μm filtered PBS. A 130 μL aliquot of concentrated EVs was taken, diluted to 600 μL with 0.22 μm filtered PBS and named “UF-pellet”. Another 130 μL aliquot was used for SEC.

### 4.5. Size-Exclusion Chromatography

SEC was performed using qEV single 70 nm SEC columns (Izon Science Ltd, Burnside, New Zealand). Prior to use, the storage buffer was removed and the column was washed with 5 mL of 0.22 μm filtered PBS. After inserting the 130 μL sample, eight fractions were collected. The volume of the first fraction was 400 μL and fractions 2–8 were 600 μL each. One 100 μL aliquot from each “pellet” sample and fractions 2–8 was stored at −70 °C for further analyses. The remaining 500 μL was mixed with 2.5 mL of Qiagen Qiazol lysis reagent (#79306, Qiagen, Venlo, The Netherlands), kept at RT for 5 min and stored at −70 °C prior to RNA isolation.

### 4.6. Nanoparticle Tracking Analysis

The particle concentration and size distribution from pellets and individual SEC fractions (2–8) was measured using a NanoSight NS300 (Malvern, Worcestershire, UK). The 100 nm latex beads (#NTA4088, Malvern Pananalytical, Malvern, United Kingdom) were used as a positive control and 0.22 μm filtered PBS as a negative control. Each sample was diluted 1:50 or greater to obtain a concentration between 5 × 10^7^–9 × 10^8^ particles/mL. Each 400 μL sample was injected into the sample chamber at a constant flow rate using the Malvern NanoSight syringe pump system. For all recordings, the camera level was adjusted to 15, and three 60 s videos were recorded for each sample. For analysis, the detection threshold was set to 5 and the other settings were “automatic”.

### 4.7. Protein Concentration

Protein concentration of pellets and SEC fractions were measured using the Pierce™ BCA Protein Assay Kit (#23225, Thermo Fisher Scientific, Waltham, MA, USA) according to the manufacturer’s instructions. The reliable detection limit of the kit is 20 μg/mL; therefore, samples with a protein concentration between 10–20 μg/mL were assigned an arbitrary value of 15 μg/mL and those with concentrations below 10 μg/mL were assigned an arbitrary value of 5 μg/mL.

### 4.8. Transmission Electron Microscopy

Copper-carbon film grids (400 mesh; EM Resolutions, Sheffield, United Kingdom) were glow-discharged using a Quorum K100X glow discharger. Grids were then placed on a 5 μL droplet of sample (on dental wax) for 2 min. Buffer salts were removed by transferring the grids twice to a fresh drop of distilled water and incubated for 5 s each. The excess fluid was removed with filter paper and the grids were transferred to one drop of uranyl acetate (1.5% in distilled water) and incubated for 1 min. Excess fluid was removed with filter paper and the grids were air dried prior to imaging. Grids were imaged using an FEI/ThermoFisher Scientific Tecnai G2 TEM electron microscope run at 200 keV accelerating voltage and using a 20 μm objective aperture to improve contrast. Images were acquired with an ORCA HR high resolution CCD camera using a Hamamatsu DCAM board and running the Image Capture Engine, software version 600.323, from AMT Corp. (Advanced Microscopy Techniques Corp. Danvers, USA).

### 4.9. RNA Isolation and Analysis

RNA analysis was performed from each “pellet” sample, fraction 2 (pre-EVs), fraction 3 (EV fraction), combined fractions 4 + 5 (middle fractions) and combined fractions 6–8 (protein fractions). Qiazol-sample mixes were defrosted on ice, combined with 0.2 volume of chloroform and left for 5 min at RT. Solutions were centrifuged (12,000× *g*, 15 min, 5 °C), to allow separation and collection of the aqueous phase, which was in turn combined with 2.5 volume of 96% ethanol. The sample mix was added to miRNeasy Mini Kit columns (Qiagen, Hilden, Germany, #217004) and washed according to the manufacturer’s instructions. Finally, the total RNA was eluted in 30 μL of nuclease free water. The small RNA profile of isolated RNAs was measured from 1 μL of sample using the Agilent Bioanalyzer Small RNA kit (#5067-1548, Agilent Technologies, Santa Clara, CA, USA). The concentration of 10–40 nt and 40–80 nt small RNA were calculated using the 2100 Expert software with custom ranges.

### 4.10. Western Blotting

Samples were mixed with 4× Pierce™ LDS Sample Buffer (#84788, ThermoFisher Scientific, Waltham, MA, USA) including DTT (0.3M) and denatured for 5 min at 75 °C. For Western blot analysis, 5 μL (fractions 6–8 in UF+SEC) or 25 μL (other samples) of protein sample was separated using a 4–20% SDS-PAGE (Mini-PROTEAN^®^ TGX Stain-Free™ Protein gels, #4568094, BIO-RAD, Hercules, CA, USA) and MOPS running buffer and transferred to nitrocellulose membranes using a BIORAD Trans-Blot SD semi dry transfer system. Membranes were blocked for 45 min (PBS, 0.1% Tween 20, 5% dried milk) and primary antibodies against Tsg101 (ab83, Abcam, Cambridge, UK), Alix (ab117600, Abcam, Cambridge, UK), Tamm-Horsfall protein (uromodulin, ab733, Millipore, Burlington, MA, USA) and CD9 (#MA1-80307, Invitrogen, Carlsbad, CA, USA) were used at 1:1000 dilution o/n at 4 °C. Horseradish peroxidase (HRP)-conjugated secondary antibodies (rabbit anti-goat (#P0160, Dako, Agilent Technologies, Santa Clara, CA, USA, 1:5000) and rabbit anti-mouse (#P0260, Dako, 1:5000)) were used and chemiluminescence activated using the Amersham ECL Prime detection reagent kit (#RPN2232 GE Healthcare, Chicago, IL, USA). Bands were visualised using the signal accumulation mode with a Bio-Rad ChemiDoc™ MP System (Bio-Rad, Hercules, CA, USA).

### 4.11. Statistical Analyses

Statistical analyses were performed using GraphPad Prism 8 for macOS (San Diego, US), version 8.2.1 (279). For normalised particle numbers and particle/protein ratio were analysed using the Friedman test followed by uncorrected Dunn’s test. For comparison of the percentage of total small RNA of size 10–40 nt and 40–80 nt present within fractions 3 and 6–8, the Wilcoxon matched-pairs signed rank test was used. The ratio of 10–40 nt sized small RNA was calculated for samples from UF and the ratio of 40–80 nt sized small RNA samples was calculated from all methods combined. *p*-values ≤ 0.05 were considered statistically significant.

### 4.12. EV-TRACK

We have submitted all relevant data of our experiments to the EV-TRACK knowledgebase (EV-TRACK ID: EV210150) [52].

## Supplementary Materials

The following are available online at https://www.mdpi.com/article/10.3390/ijms22094881/s1, Figure S1: SDS-PAGE profiles of pellets and SEC EV fractions, Figure S2: Western blot analysis from round B and C and Figure S3: Effect of an additional slow-speed centrifugation on the small RNA profile.
