# Supplementary material for: Size-Exclusion Chromatography Separation Reveals That Vesicular and Non-Vesicular Small RNA Profiles Differ in Cell Free Urine
Source: Int J Mol Sci. 2021 May 5;22(9):4881. doi: 10.3390/ijms22094881 (PMC8124894; doi:10.3390/ijms22094881)

Size-exclusion chromatography separation reveals distinct vesicular and non-vesicular small RNA profiles in cell free urine

Jenni Karttunen, Sarah E Stewart, Lajos Kalmar, Andrew J Grant, Fiona E Karet Frankl and Tim L Williams

Corresponding author: Tim L Williams, Department of Veterinary Medicine, University of Cambridge, CB3 0ES, UK. Email tlw27@cam.ac.uk

Supplementary Figure S1. SDS-PAGE profiles of pellets and SEC EV fractions

Protein profiles from samples in round B imaged with TGX Stain-Free gel system. Samples include EVs concentrated with three methods before ("pellet") and after further purification with SEC. Before the SEC, the profiles were different between the three methods indicating the presence of non-EV related proteins. After SEC the protein profiles were similar with all three methods indicating equally pure vesicles. Apart from the "UF pellet" sample (1:10 dilution), an equal volume of sample was loaded in the gel. UF=ultrafiltration, PR=precipitation, UC=ultracentrifugation, SEC=size-exclusion chromatography.

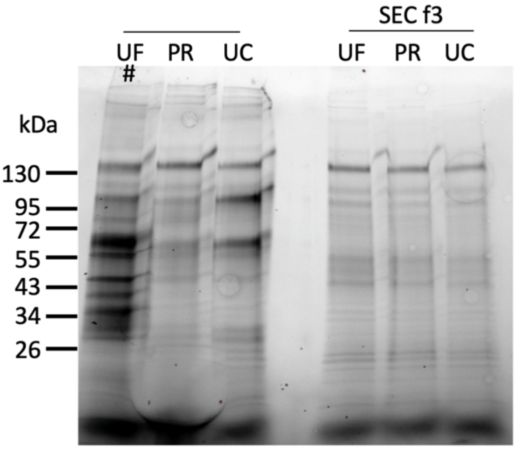

1:10 dilution compared to other samples

Supplementary figure S2. Western blot analysis from round B and C

Western blot analysis of rounds B and C with samples of EVs concentrated with three methods before ("pellet") and after further purification with SEC. After SEC, UF had the highest intensity confirming the highest number of vesicles measured with other methods. Between three rounds, round C had the lowest number of vesicles and a weaker signal was also evident with western blot analysis. UF=ultrafiltration, PR=precipitation, UC=ultracentrifugation, SEC=size-exclusion chromatography.

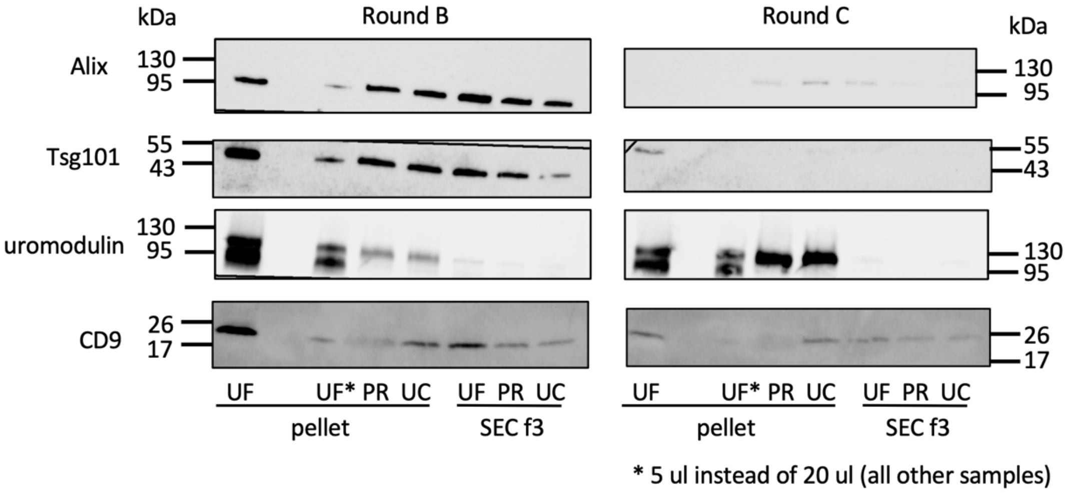

Supplementary figure S3. Effect of an additional slow-speed centrifugation on the small RNA profile

This experiment aimed to evaluate the effect of low-spin centrifugation on the small RNA profile of fresh urine. For individual replicates (n=3, A-C), a first void urine sample from an individual donor was collected and a protease inhibitor was added. The urine sample was divided into two aliquots and the first aliquot was centrifuged at a low-speed (10 min at 1000g, +5C) to eliminate cells. Both of the aliquots were then centrifuged for 20min at 17k xg and filtered with 220nm filter. Equal volumes of the filtered urine from both aliquots were concentrated with Amicon ultra concentrator columns and adjusted to 300ul. 130 ul of the concentrated urine was further purified with SEC and another 130ul was diluted to 600ul as "pellet" sample. 500ul of each sample was used in RNA isolation, fractions 6-8 were combined for protein fractions. Small RNA profile was measured with Agilent Bioanalyzer Small RNA chip. No difference was detected in the smallRNA profiles between samples with and without the additional low-speed centrifugation.

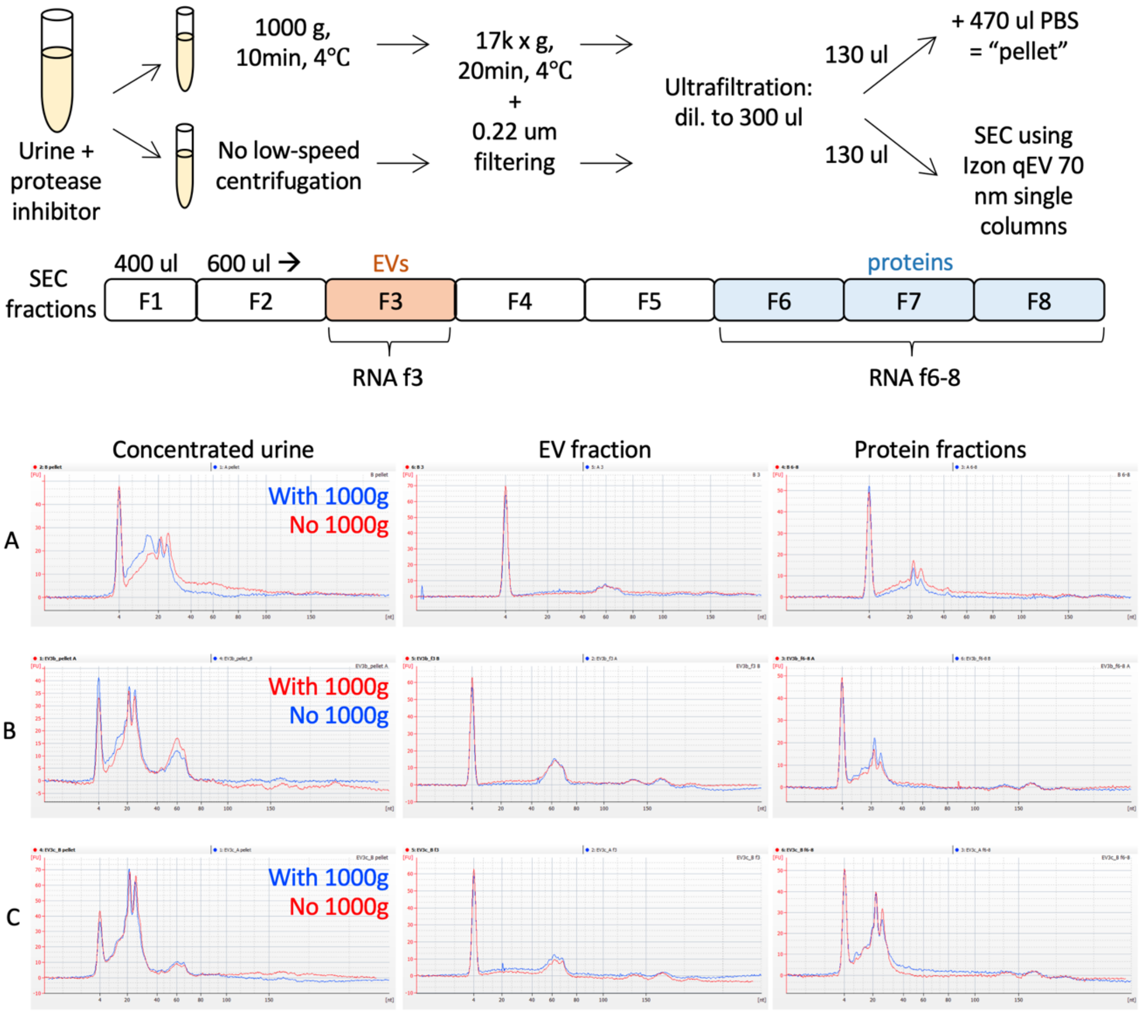

Supplement: Supplementary file 1 [file ijms-22-04881-s001.zip › ijms-1164856-supplementary.pdf]
